# Investigation of *Brassica* and its relative genomes in the post-genomics era

**DOI:** 10.1093/hr/uhac182

**Published:** 2022-08-25

**Authors:** Jian Wu, Jianli Liang, Runmao Lin, Xu Cai, Lei Zhang, Xinlei Guo, Tianpeng Wang, Haixu Chen, Xiaowu Wang

**Affiliations:** Institute of Vegetables and Flowers, Chinese Academy of Agricultural Sciences, 100081 Beijing, China; Institute of Vegetables and Flowers, Chinese Academy of Agricultural Sciences, 100081 Beijing, China; Institute of Vegetables and Flowers, Chinese Academy of Agricultural Sciences, 100081 Beijing, China; Institute of Vegetables and Flowers, Chinese Academy of Agricultural Sciences, 100081 Beijing, China; Institute of Vegetables and Flowers, Chinese Academy of Agricultural Sciences, 100081 Beijing, China; Institute of Vegetables and Flowers, Chinese Academy of Agricultural Sciences, 100081 Beijing, China; Institute of Vegetables and Flowers, Chinese Academy of Agricultural Sciences, 100081 Beijing, China; Institute of Vegetables and Flowers, Chinese Academy of Agricultural Sciences, 100081 Beijing, China; Institute of Vegetables and Flowers, Chinese Academy of Agricultural Sciences, 100081 Beijing, China

## Abstract

The Brassicaceae family includes many economically important crop species, as well as cosmopolitan agricultural weed species. In addition, *Arabidopsis thaliana,* a member of this family, is used as a molecular model plant species. The genus *Brassica* is mesopolyploid, and the genus comprises comparatively recently originated tetrapolyploid species. With these characteristics, *Brassica*s have achieved the commonly accepted status of model organisms for genomic studies. This paper reviews the rapid research progress in the Brassicaceae family from diverse omics studies, including genomics, transcriptomics, epigenomics, and three-dimensional (3D) genomics, with a focus on cultivated crops. The morphological plasticity of Brassicaceae crops is largely due to their highly variable genomes. The origin of several important Brassicaceae crops has been established. Genes or loci domesticated or contributing to important traits are summarized. Epigenetic alterations and 3D structures have been found to play roles in subgenome dominance, either in tetraploid Brassica species or their diploid ancestors. Based on this progress, we propose future directions and prospects for the genomic investigation of Brassicaceae crops.

## Introduction

Brassicaceae, often called Cruciferae or the mustard family, comprises 4636 known species in 340 genera [1]. Many of them have been domesticated as important crops for agriculture, ornaments, or condiments, some of which are also of medicinal significance. This family includes species with both ancient and recent polyploidies, as well as species with relatively small genomes, such as the model plant species *Arabidopsis thaliana*. A key agricultural genus of the Brassicaceae family is the *Brassica* genus, in which the six most commonly known members are three diploid species, *Brassica rapa* (A genome, *n* = 10), *Brassica nigra* (B genome, *n* = 8), and *Brassica oleracea* (C genome, *n* = 9), and three allotetraploid species, *Brassica juncea* (AB genome, *n* = 18), *Brassica napus* (AC genome, *n* = 19), and *Brassica carinata* (BC genome, *n* = 17). The genomic relationships of these six representative members have been defined as the ‘Triangle of U’.

**Figure 1:**
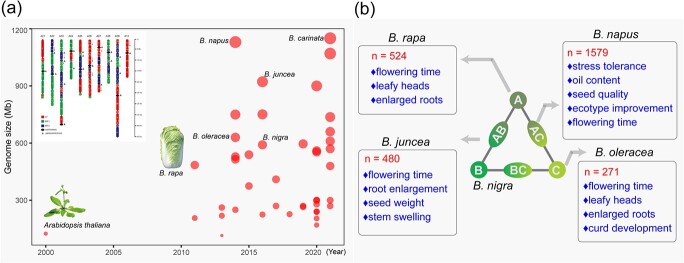
Overview of genomic studies in Brassicaceae. Studies on the whole-genome sequencing of Brassicaceae species. **a** Non-redundant statistics of sequenced species in Brassicaceae. The red dot represents each sequenced species, varying in size according to the genome size. The details of these sequenced species are provided in Table S1 (see online supplementary material). The image in the upper-left corner shows the composition of three subgenomes in mesopolyploid *Brassica rapa* [10]. **b** Studies on important agronomic traits in *Brassica* crops using population-scale resequencing strategies. *n*, total number of resequenced accessions within each species.

With the rapid advances in sequencing technology, more and more Brassicaceae species have been sequenced and assembled into high-quality reference genomes. Using the latest statistics from 2022, 43 species in Brassicaceae have been sequenced (https://plabipd.de/timeline_view.ep) (Fig. 1a, Table S1, see online supplementary material). After the genome assembly of the first Brassicaceae species, *A. thaliana*, it took more than 10 years for the second one, *B. rapa* [2], to be sequenced in 2011. The release of the *B. rapa* genome sequence not only warrants further analysis of gene functions within *B. rapa*, but also provides an important reference for the study of the polyploidization of Brassicaceae species and the evolution of members of the ‘Triangle of U’. After this, it took another 10 years for all the other members of the ‘Triangle of U’ to be sequenced [3–8]. In the last five years, a quickly increasing number of genomes of species from Brassicaceae have been decoded. Some representative species, e.g. *B. napus*, *B. oleracea*, and *B. rapa*, have obtained high-quality reference genomes following multiple rounds of genome upgrades [9–12], while some less popular species have also recently been decoded [13–24] (Table S1, see online supplementary material).

The release of these reference genomes has bolstered the exploration of genomic variation, modification, and regulation in Brassicaceae species. During the past five years, the major *Brassica* crops have been resequenced, scaling up to populations of several hundred accessions. Moreover, investigations using whole-genome bisulfite sequencing (WGBS), high-throughput chromatin conformation capture (Hi-C), chromatin immunoprecipitation assays with sequencing (ChIP-seq), Assay for Transposase-Accessible Chromatin using sequencing (ATAC-seq), and DNA Affinity Purification and sequencing (DAP-seq) have been reported for Brassicaceae plants. These have significantly improved our cognizing of the genome modification and regulation of gene expression. Recently, *de novo* sequencing of multiple accessions of several *Brassica* crops has brought investigations of genomic variation into the pan-genomics era, and RNA sequencing (RNA-seq) has evolved to explore single-cell and spatial transcriptomes. Here, we summarize the recent progress of genomics investigations of Brassicaceae species, with a focus on their application in cultivated crops.

## Variation in Brassicaceae genomes

*A. thaliana* has been taken as a model organism for basic plant research. Intraspecific genome variation study on a large scale started at the beginning of 2008 by launching the 1001 Genomes Project as a pioneer for plant species-wide diversity research [25]. The aim of the project was to describe detailed whole-genome sequence variation in at least 1001 accessions of *A thaliana*. A variation map derived from resequencing of 1135 *A. thaliana* accessions was published in 2016 [26]*.* As important cultivated crops, *Brassica* and *Raphanus* species have received significant attention in the characterization of genomic variations. With the exception of *B. carinata*, all *Brassica* crops have been subjected to large-scale genome resequencing in different laboratories [27–31], while pan-genomes have been constructed for *B. napus*, *B. oleracea*, *B. rapa,* and *Raphanus* [11, 27, 32, 33]. The in-depth analysis of whole-genome variation data has provided not only the genomic features explaining the domestication of extreme morphotypes but has also identified specific genes and variants contributing to important agronomic traits.

## Genomic variation revealed by population-scale resequencing in *Brassica*

Due to multiple rounds of genome duplication, *Brassica* species show unique genomic variations. In the past five years, hundreds of accessions within each of the *Brassica* crops have been resequenced (Fig. 1b). The genomes of the three diploid members of the ‘Triangle of U’ were all derived from an extra whole-genome triplication (WGT) of the tPCK ancestors, which led to three subgenomes, namely, Least Fractionated (LF), More Fractionated (MF1), and Most Fractionated (MF2). While LF is the least variable subgenome, the WGT increased the genomic variation contributing to the diversified morphotypes in *Brassica* species [34]. During the intraspecific diversification of the A and C genomes, it was found that the gene repertoire, transposable element (TE) content, and the number of variations varied greatly among individuals [27, 33]. This phenomenon has also been observed in the allotetraploid species *B. napus* (AACC) and *B. juncea* (AABB) [29, 31]. Investigations in both *B. napus* and *B. juncea* established the same conclusion: A subgenomes in both allotetraploid species have a higher frequency of genetic recombination and maintain higher nucleotide diversity than either the C or B subgenomes. However, the mechanism is yet to be clarified.

*Brassica* crops have been domesticated to a number of extreme morphotypes, such as the leafy heads in Chinese cabbage (*B. rapa* ssp. *pekinensis*) and cabbage (*B. oleracea var. capitata*); enlarged roots in turnip (*B. rapa ssp. rapa*), rutabaga (*B. napus* var. *rapifera*), and root mustard (*B. juncea* ssp. *napiformis*); enlarged stems in kohlrabi (*B. oleracea* var. *gongyloides*) and tuber mustard (*B. juncea* var. *tumida*); and thickened inflorescences in cauliflower (*B. oleracea var. botrytis*) and broccoli (*B. oleracea var. italica*). In 2016, the domestication of leafy-head and tuber-forming *Brassicas* was investigated by resequencing 199 *B. rapa* accessions and 119 *B. oleracea* accessions [28]. In that study, not only selection sweeps of the domestication of leafy heads and root/stem enlargement were identified but also candidate genes for these traits were pinpointed. Moreover, homoeologous genes were selected in parallel in both species during trait domestication for leafy head and root/stem enlargement*.* Cai *et al.* genotyped structure variations (SVs) across 524 diverse *B. rapa* accessions and found that four SV-containing genes (*BrPIN3.3*, *BrMYB95.3*, *BrFL5.1*, and *BrSAL4.2*) might be involved in the formation of leafy heads [27]. Very recently, Sun *et al.* identified two genes with chloroplast-related functions that are responsible for the yellow leaves traits using phenotype screening and resequencing of a large-scale ethyl methane sulfonate (EMS) mutant population [35]. This study demonstrated that the strategy of combination phenotypic and genotypic screening is powerful in the detection of candidate genes for target traits.

By analysing selection signatures for root mustard in *B. juncea,* 14 candidate genes were identified as being involved in the storage roots formation. These genes include *CDC48A4* and *EXP1* genes and genes involved in cell division, cell expansion, and the regulation of auxin signaling. This research also highlighted the subgenomic prevalence of selective sweeps in the Aj subgenome (A subgenome in *B. juncea*) over the Bj subgenome (B subgenome in *B. juncea*) [29]. Cauliflower curd composes of thousands of inflorescence meristems with floral arrested. By analysing resequencing data of 104 accessions of cauliflower and 167 accessions of the other morphotypes of *B. oleracea*, Guo *et al.* detected dozens of selected SVs and associated genes that are potentially involved in the curd formation and enlargement [36]. Additionally, variants associated with seed yield, oil content, erucic acid, and glucosinolates in seeds, as well as seed weight, were also revealed in oil *Brassica* crops (Fig. 1b).

Genome-wide association analysis (GWAS) is a widely used approach for exploring the sequence variants associated with complex traits in crops. The *Brassica* 60 k SNP array has been widely used to genotype *B. napus* natural populations in GWAS research [37–40]. Very recently, based on large-scale genome resequencing of 403 diverse rapeseed accessions, Hu *et al.* traced the genomic basis of agronomic traits during modern rapeseed breeding. A total of 628 causative candidate genes were identified for 56 agronomic traits in *B. napus* by GWAS [41]. Although GWAS is powerful in genome-widely detecting of genetic variations associated with target traits, the resolution of GWAS is generally not sufficient to directly determine the causal genes. Therefore, the combination of GWAS and transcriptome-wide association studies (TWAS) provides more powerful approaches for genetic dissection of target traits. Similarly, Tang *et al.* developed a gene prioritization framework based on multi-omics data and information from *A. thaliana* to prioritize the causal gene *BnPMT6* for seed oil content in *B. napus* [42].

Flowering time, as one of the most important agronomic traits, has been a specific focus in investigations of trait domestication or GWAS analyses in *Brassica* crops. Su *et al.* (2018) resequenced 194 Chinese cabbage accessions representing spring, summer, and autumn ecotypes [43]. They identified that the sequence variations in the *cis* elements of the *BrVIN3.1* promoter contribute to varied vernalization responses in different ecotypes of Chinese cabbage [43]. Wu *et al.* resequenced 991 *B. napus* accessions and found that single nucleotide polymorphisms (SNPs) in the promoter regions of *FT* and *FLC* orthologs specifically in line with the three rapeseed ecotype groups [31]. Similarly, in *B. rapa*, a nonsynonymous mutation at the 58th nucleotide of exon1 and a splicing site mutation in intron 6 of *BrFLC1* contributed to flowering time variations [44, 45]. In *B. juncea*, two SNPs in *SRR1* and five SNPs in *VIN3* were identified as being closely associated with flowering time [29] using population-scale resequencing strategies.

## Pan-genomes are the new references for mining genomic variations

**Figure 2:**
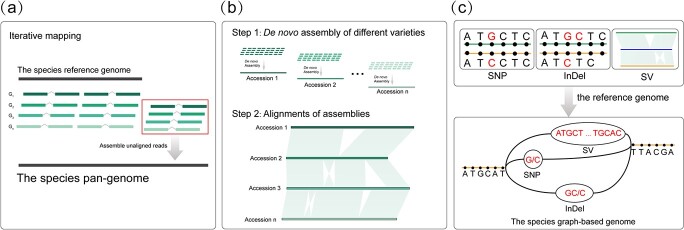
Strategies commonly used for mining genomic variations in the pan-genome era. **a** Construction of a pan-genome using an iterative mapping approach. **b** Construction of a pan-genome by assembling the genomes of diverse varieties. **c** Construction of a pan-genome by integrating genomic variations and the reference genome.

The pan-genomes of important Brassicaceae crops have been constructed using three popular approaches. The first involves aligning reads from a sequenced accession onto the reference genome and then assembling the unaligned reads into novel contigs (Fig. 2a). This ‘map-to-pan’ strategy was employed in constructing the *B. oleracea* pan-genome [33]. The second is *de novo* assembly of the genomes of diverse varieties, while the third is to construct a species graph-based genome (Fig. 2b). The pan-genome of *A. thaliana* [46] was constructed using the second strategy, while the pan-genomes of *Raphanus* [32] and *B. rapa* [27] were *de-novo* assembled using diverse varieties and a graph-based genome strategy (Fig. 2c). The iterative assembly for constructing the pan-genome is cost-effective, as an iterative assembly fills up gene sequences that are absent in the reference genome, and the accessions are sequenced on a low-cost short-read sequencing platform. However, a pan-genome constructed entirely using short reads largely limits the exploration of complex structural variations. In recent years, the development of long-read sequencing and graph-based genome strategies has resolved this limitation, and species are moving toward population-scale long-read sequencing [47, 48].

Pan-genome analysis can reveal hidden genomic variations. As early as 2016, the *B. oleracea* pan-genome was published by assembling short reads [33]. It was found that 18.7% of genes in the pan-genome were composed of dispensable genes, including genes related to major agronomic traits, such as disease resistance, flowering time, etc. [33]. Long-read sequencing technologies were employed for constructing the pan-genomes of *A. thaliana* [46], *B. rapa* [27], and *Raphanus* [32], revealing more hidden genomic variations in these three species. For example, the *A. thaliana* pan-genome constructed by assembling seven *A. thaliana* accessions revealed that ~1900 genes were absent from the reference genome [46]. Zhang *et al.* (2021) constructed a pan-genome of *Raphanus* that included 11 accessions from domesticated, wild, and weedy radishes [32]. In the *Raphanus* pan-genome, the number of SVs is ~26 × 10^3^ per sample, which is similar to the number in soybean pan-genome [32]. However, the size of the radish genome is only half of that of the soybean genome, and the SV density of *Raphanus* is twice that of the soybean genome. Cai *et al.* (2021) constructed a pan-genome consisting of 18 *B. rapa* accessions from six morphotypes. It revealed that each genome contains 15.14%–37.39% of sequences that were not syntenic with the Chiifu reference genome. A total of 33.24–56.7 Mb insertions and 35.75–58.84 Mb deletions (size ≥50 bp) were detected in the *B. rapa* pan-genome. Further analysis indicated that SV highly associated to the morphotype domestication in *B. rapa* [27]. Transposable elements (TEs) are major components of eukaryotic genomes. Recently, Cai *et al.* (2022) developed a novel pipeline to detect TE insertion polymorphisms (TIPs) on a population scale via combination of the *B. rapa* pan-genome and resequencing data from 524 *B. rapa* accessions and revealed that the TIPs in TIP-containing genes had been selected more strongly than non-synonymous SNPs [49]. In summary, the graph-based pan-genomes of Brassicaceae species will serve as a useful reference for GWAS or domestication analysis at the SV level, providing significant advantages over SNP-based analysis using a single genome as the reference.

In addition, *Brassica* genus-wide pan-genomes were established. The first and foremost advantage of the genus-wide pan-genomes is facilitating gene content description under a framework using systematic nomenclature proposed by the Multinational Brassica Genome Project [50]. One of the unresolved questions in the evolution of *Brassica* genomes is the mechanism by which LF subgenome exhibits less gene loss (fractionation) than the MF1 and MF2 subgenomes. It has been proposed that *Brassica* mesohexaploidy had occurred via a two-step process, according to the gene density in the three subgenomes of *B. rapa* [2]. The different methylation levels between the LF and MF subgenomes further supported this hypothesis [51]. The genus-wide pan-genomes for *Brassica* enabled updating Ancestral Crucifer karyotype (ACK) block organization, and provided further evidence of two-step pathways in the *Brassica* genome evolution.

## Homoeologous exchanges as important resources for generating novelties in *brassica* allopolyploids

Homoeologous exchanges (HEs) specifically describe the exchanges of chromosome segments in allopolyploids, which occur from crossover formation between homoeologous genomes [52, 53]. They have been suggested as important resources for increasing genomic variations and generating new phenotypes to be selected for domestication in allopolyploid species. Homoeologous exchanges can result in one of the homoeologous DNA fragments becoming fixed over the other, consequently leading to copy number variation (CNV) or presence-absence variation (PAV) of the genes [54, 55]. HEs have been reported as prevalent events in many allopolyploid species, including rapeseed [3], peanut [56], bread wheat [57], polyploid rice [58], and other wild species [59]. Among these species, many in-depth studies regarding HE mechanisms and impacts on phenotypes have been conducted on *B. napus.* The HE phenomenon was first reported in synthetic lines, which extended back to at least 1995 [60], and later, the association of HE with flowering time diversification after several generations was also established [52, 61–64]. Recently, owing to the rapid development of the genomic era, many studies have been conducted at a large population scale and have discovered that HEs have a much higher frequency in different domesticated varieties than expected [64–66]. Additionally, HEs have been identified as the major cause of gene PAV in different populations [54], and their impacts on gene expressional changes were demonstrated to be proportional to the gene copy number changes [53, 54]. The genes affected by HEs have also been shown to be responsible for many important trait diversities in *Brassica* polyploids, including flowering time [55, 67], leaf morphology [63], seed glucosinolates content [64], and disease resistance [54], suggesting the potential role of HEs in generating phenotypic novelties for domestication.

Some important genes responsible for HE have been reported in *B. napus*. Gonzalo *et al.* showed that reducing *MSH4* copy number prevents meiotic crossovers between non-homologous chromosomes in *B. napus* [68]. Recently, by applying both quantitative trait locus (QTL) mapping and cytogenetic analysis in a resynthesized segregating *B. napus* population, Higgins *et al.* (2021) successfully identified several important quantitative loci for controlling HEs. The major locus *BnaPh1* on chromosome A9 contributed 32–58% observed variation of homoeologous recombination, and the genes found in the locus would facilitate the identification of the causal and new genes for controlling successful meiotic adaptation in polyploids [69]. Compared to the artificially synthesized *B. napus,* natural *B. napus* plants have much lower frequency of HEs. Reports on genes that reduce HE in *B. napus* suggest that natural allopolyploid plants have undergone selection to reduce the frequency of HE. Further studies on HEs and their mechanisms could ultimately offer new breakthroughs for improving diversity in polyploid crops [70, 71].

## Domestication of *Brassica* crops revealed by genomics analysis

*Brassica* comprises about 35 species of mainly annual herbs, with some perennial herbs and small shrubs. Cultivated *Brassica*s are not only used for different purposes, such as fresh and preserved vegetables, vegetable oils, and condiments, but are also cultivated worldwide in different climatic conditions. Moreover, *Brassica* species possess high genome plasticity, allowing for the domestication of crops with extremely high morphological variability. Similar traits, such as the traits of enlarged root/stem and leafy head, have been domesticated in different *Brassica* species in parallel. All of these features render *Brassica* an ideal system for investigating crop domestication and artificial selection.

**Figure 3:**
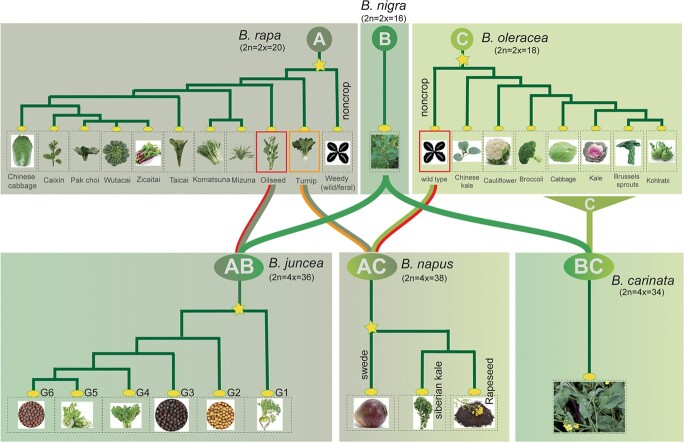
Crop domestication in *Brassica*. ‘A’, ‘B’, ‘C’, ‘AB’, ‘AC’, and ‘BC’ represent the genomes of *Brassica rapa*, *B. nigra*, *B. oleracea*, *B. juncea*, *B.* napus, and *B. carinata*, respectively. The phylogenetic relationships of *Brassica* crops were based on information published previously by McAlvay *et al.* [72], Cai *et al*. [27], Cheng *et al.* [28], Lu *et al.* [30], Yang *et al.* [5], and Kang *et al.* [29]. The stars represent the boom of morphotypes in target species. Background colors of A, B, and C genomes are olive green, green, and grass green, respectively; the background colors of AB, AC, and BC genomes are the gradient background colors representing their progenitors.

It is widely accepted that turnip was the first domesticated *B. rapa* crop type [28, 72]. Previously, Song *et al.* [73] proposed that Europe was the primary center of domestication for turnip and turnip rape, while China was the second center where various Asian vegetable crops, including Chinese cabbage, pak choi, wutacai, mizuna, and komatsuna, were domesticated (Fig. 3). Recently, by analysing the evolution of *BrFLC1* in diverse *B. rapa* crops, it was found that the ancient crops (e.g. turnip, turnip rape) were skewed toward carrying the haplotype of late flowering; the crops that differentiated in China were biased toward carrying the early flowering haplotype, supporting two independent centers of origin of *B. rapa* [44]. *B. oleracea* has been domesticated into even more extreme morphotypes (Fig. 3). Previously, various origins of cultivated *B. oleracea* have been proposed, including a single origin from wild *B. oleracea* in western Europe, as well as triple and even multiple origins involving related wild species [74]. Recently, Mabry *et al.* proposed that the Aegean endemic *B. cretica* is the closest living relative of cultivated *B. oleracea*, supporting a single origin of cultivation in the Eastern Mediterranean region [75]. Leaf heading and enlarged root/stem traits were domesticated respectively in *B. rapa* and *B. oleracea* in parallel. One study proved that parallel selection at the subgenome level played a critical role in the domestication of parallel traits in these two different species [28].

Based on the ‘Triangle of U’ model, the pairwise hybridizations of *B. rapa* and *B. nigra* formed *B. juncea*, of *B. rapa* and *B. oleracea* formed *B. napus*, and of *B. nigra* and *B. oleracea* formed *B. carinata*. Establishing the comparative genomics platform for the species of the ‘Triangle of U’ allowed the examination of the dynamics of polyploid evolution and the impact of subgenome dominance in domestication and agronomical improvement. However, as each of the ancestor species possesses diverged morphotypes, the most direct ancestor morphotype has yet to be defined for each of the allotetraploid species. A recent genomics investigation supported that the Aj subgenome (A subgenome in *B. juncea*) was derived from *B. rapa* ssp. *tricolaris* [29], and the An subgenome (A subgenome in *B. napus*) was derived from European turnip (Fig. 3) [5]. This implicates that *B. juncea* and *B. napus* evolved from independent geographical origins, as their progenitor *B. rapa* ssp. *tricolaris* was distributed in Asia, while European turnip was distributed in Europe. As for the Cn subgenome (C subgenome in *B. napus*), Lu *et al.* proposed that it is derived from the common ancestor of kohlrabi, cauliflower, broccoli, and Chinese kale [30]. By analysing 480 *B. juncea* accessions collected from 38 countries, Kang *et al.* proposed that West Asia is most likely to be the single origin of *B. juncea*. Subsequently, various *B. juncea* vegetable and oil crops formed through spontaneous gene mutations and introgressions along three independent routes of eastward expansion [29]. It was proposed that *B. napus* was formed ~7500 years ago [3] via natural interspecific hybridization of European turnip and wild *B. oleracea* (Fig. 3). Subsequently, the three ecotypes of winter, spring, and semi-winter formed. It was reported that winter *B. napus* was first domesticated in Europe [76]. Spring *B. napus* was developed in Europe and spread to England [77], and the semi-winter ecotypes were mainly cultivated in China via introduction from Europe [30, 78]. In the case of *B. carinata*, the relationship between the Bc and Cc subgenomes is greater than that between other two tetraploid subgenomes (Bj and Cn) and their respective diploid parents [6].

## Epigenetics and 3D structure of Brassicaceae genomes

Over the past decades, plant linear genomes and epigenetic modifications have been studied extensively. More recently, three-dimensional (3D) genome structures in plants began to be rapidly unveiled. Accumulating evidence has revealed that not only epigenetic modification in the linear DNA sequence but also 3D genome architecture play an important role in determining genome organization, genome functionality, and gene expression regulation [79]. With abundant genetic resources and updated reference genome sequences in Brassicaceae plants, advances in sequencing technology and multidisciplinary methods facilitated the study epigenetic regulation ad 3D genome architecture and their relationships with genome complexity and subgenome dominance in Brassicaceae.

## Epigenetics of Brassicaceae genomes

Epigenetics is the study of any potentially stable and heritable change in gene expression or cellular phenotype that does not involve the changes in DNA sequence [80]. The underlying mechanisms of epigenetic regulation are mediated by DNA methylation, histone modification, non-coding RNA, etc. In the 1001 Epigenomes Project of *A. thaliana*, 1107 high-quality single-base resolution methylomes and 1203 transcriptomes of *A. thaliana* have been established [81]. It revealed that geographic origin is highly related to genome-wide DNA methylation levels and altered gene expression caused by epialleles, although the genetic basis of methylation variation is highly complex [81].

The *Brassica* genus represents a fascinating model for epigenetic studies because of its unique genomic variations [2, 51]. To date, many epigenetic studies on *Brassica* have been conducted not only at the subgenome level but also across duplicated gene copies. These studies suggest that epigenetic modifications may be the determinants of the subgenome dominance and functional diversification of duplicated genes. In diploid *Brassica*, global analysis of the DNA methylation profile showed that the DNA methylation level was similar or higher in *B. rapa* compared to that in *B. oleracea* but was much higher than that of *A. thaliana,* probably resulting from the difference in genome structure, such as the difference in the amount and distribution of TEs and repeat sequences [82, 83]. The three subgenomes of *B. oleracea* show imbalanced DNA methylation, with the dominant LF subgenome exhibiting the lowest levels of DNA methylation. Moreover, the triplicated gene copies appear to have independent DNA methylation patterns, and the non-syntenic genes have significantly enhanced DNA methylation [84]. In *B. rapa*, the single-copy retained genes were found to have significantly higher DNA methylation compared to those of genes retained in pairs or triplets [83]. Generally, the gene expression level is negatively associated with DNA methylation. These results suggest that DNA methylation variations in *Brassica* play a role in subgenome dominance and biased gene retention and the expressional diversification of duplicated genes [84].

Genome-wide profiling of histone H3 lysine methylation in *B. rapa* found that H3K4me3 and H3K36me3 are enriched in the transcription start sites [85]. Genes with H3K4me3 and H3K36me3 marks have higher expression levels but a low degree of tissue specificity [85]. In contrast, H3K27me3 marks are correlated with decreased or low gene expression or high tissue-specific gene expression [86, 87]. Furthermore, the distribution of H3K36me3 and H3K27me3 vary between homoeologous paired genes, which result in their variations in gene expression levels or tissue specificity, and eventually result in their sub-functionalization [85–87].

Comparative analysis of TE and 24-nt small RNA in *B. rapa* showed that the distribution of TEs is imbalanced among subgenomes, which is also reflected between the flanking regions of homoeologous gene pairs. These findings suggest that the biased distribution of TEs and the targeting of 24-nt small RNAs both involved in the dominant expression phenomenon at a subgenome scale or among the homoeologous gene copies [88, 89].

In contrast to diploid *Brassica*, epigenetic alterations are thought to be involved in polyploidization events of allotetraploid *Brassica*. For example, in the *B. napus* genome, epigenetic modifications are imbalanced not only between the An and Cn subgenomes but also between homoeologous gene pairs. The Cn subgenome has a higher methylation level than that of the An subgenome, possibly resulting from higher TEs density in the Cn subgenome [3]. Comparative analysis among *B. rapa*, *B. oleracea*, and *B. napus* showed that histone H3 methylation and DNA methylation differ among these three *Brassica* species, which might be attributed mainly to differences in genome structure rather than ploidy level [90]. A recent study in *B. napus* found that the An subgenome has a higher level of active epigenetic marks and a lower level of inhibitory epigenetic marks compared with the Cn subgenome. Meanwhile, the distributions of histone modifications between homoeologous gene pairs reflect their biased expression patterns [91].

In addition, some studies on *Brassica* found that epigenetic modification such as DNA methylation played roles in genomic stability. It has been revealed that synthetic allotetraploid AACC undergoes higher DNA methylation changes than its diploid parents. This status might be highly correlated with the genomic instability of newly synthesized allotetraploid [92]. Comparative analysis of natural and synthetic *B. napus* detected that the most obvious difference in DNA methylation patterns was CHG methylation levels, which were significantly lower in synthetic rapeseed than those in natural *B. napus* [93]. Moreover, the genes related to DNA repair and nucleotide metabolism display differential expression patterns and CHG methylation levels between natural and synthetic *B. napus*, thereby suggesting that the genomic instability of newly synthesized allotetraploid plants is associated with DNA methylation changes and the disruption of the DNA repair system [93].

## 3D structure of Brassicaceae genomes

Over the past decade, the development of chromatin conformation capture (3C)-based technologies, especially Hi-C, has enabled the exploration of the hierarchical 3D structure of Brassicaceae genomes.

As reported, several types of 3D chromosomal organization, such as compartments A/B, topologically associating domains (TADs), KNOT structure, and chromatin loops, shape Brassicaceae genomes and play roles in gene expression regulation and genome function. Global analysis of high-order chromatin organization revealed that the chromatin regions of Brassicaceae could be partitioned into two compartments, namely A and B. In *Arabidopsis* and *Brassica,* the A compartment mainly overlaps with active euchromatic regions, while the B compartment mainly constitutes heterochromatin regions [94, 95]. Moreover, epigenetic modifications have been correlated with the compartmentalization of Brassicaceae genomes. For example, H3K4me2 is a typical marker euchromatin, while the distribution of H3K9me2,3 in euchromatin or heterochromatin seems to be species-specific [90]. TADs have not been detected in *A. thaliana* [94, 96, 97]. However, TADs are the most prominent feature and conserved between *B. rapa* and *B. oleracea* [95]. TAD boundaries were found to be significantly enriched in active epigenetic marks and highly transcribed genes in *B. rapa* and *B. oleracea* [95]. KNOT is the most intriguing 3D structure in the *A. thaliana* genome and has been reported to be enriched with TEs and involved in strong long-range interactions [94, 96, 97]. It was observed that Knot Engaged Elements (KEEs) or Interactive Heterochromatic Islands (IHIs) involved in KNOT structure greatly expanded in *B. rapa* but contracted in *B. oleracea* [94–96, 98]. Chromatin loops have been widely detected in plant genomes, which represent long- and short-range interactions. In *Arabidopsis*, a strong chromatin loop formation was observed between the 5′ promoter and downstream of 3′ end of the *FLC* locus, and this chromatin loop of *FLC* is disrupted after cold exposure, suggesting that the chromatin loops may be involved in the transcription of the *FLC* gene [99].

Emerging evidence suggests that 3D structure also plays significant roles in gene expression regulation, biased gene retention, and subgenome dominance in Brassicaceae genomes. Characterizing the nuclear organization of *B. rapa* and *B. oleracea*, Xie *et al.* observed that homoeologs retained on the dominant subgenome (LF) exhibited significantly stronger interaction strength than that of submissive subgenomes (MF1 and MF2) [95], which was consistent with the subgenome dominance phenomenon [51]. In addition, they also found that homoeologs retained in doublets or triplets are more likely to physically interact [95]. Furthermore, the interacting homoeolog pairs exhibited significantly higher similarity in epigenetic modifications and Gene Ontology patterns than those non-interacting homoeolog pairs in both *B. rapa* and *B. oleracea* [95]. These results suggested that the chromatin interactions of retained homoeologs are correlated with their biased retention and subgenome dominance and tend to be co-regulated with highly similar epigenetic modifications in *Brassica* [95].

It is worthwhile noting that most of the epigenetic datasets or the 3D structure in Brassicaceae were acquired from pooled tissues and only represent the average patterns of various cell types, which may ignore their dynamic changes in different cell types, leading to inaccurate results. Therefore, high-resolution single-cell strategies for epigenomic profiling or 3D structure capturing are needed to provide more specific and accurate data in Brassicaceae genomes. In addition, future studies should address the epigenomic or 3D genome structure and its functional roles in plant growth and development.

## Exploring gene expression at different levels

To explore gene expression (especially for analysing differential gene expression) at the genome scale, RNA-seq is a popular tool that is shaping our understanding of genomic function [100]. RNA-seq can capture transcriptomic dynamics during developmental stages and physiological changes under different conditions [101]. Meanwhile, RNA-seq has been driven by the development of technologies in specific niches [100]. The analysis of gene expression from traditional RNA-seq (referred to as bulk RNA-seq here) reveals the average expression of genes from bulk tissues and/or cells. Single-cell transcriptome enables the determination of gene expression at single-cell level. The spatial transcriptome may record spatial information, enabling the investigation of tissue architecture. However, some limitations in these methods should be considered. Bulk RNA-seq cannot resolve the expression of genes from special cell types, and both bulk RNA-seq and single-cell transcriptome lose the spatial contents of expressed genes. Spatial transcriptome analysis may become the routine toolkit in the future.

In the past few years, bulk RNA-seq has been widely used for studies in Brassicaceae plants. Typically, bulk RNA-seq is used to explore expression characteristics of genes at different developmental stages or under different treatments [102–109]. To explore the genetics and evolution of polyploid crops, different methods have been developed for bulk RNA-seq analysis. The subgenome dominance in *B. oleracea* was revealed by transcriptome and methylome profiling [109]. A high-density SNP linkage map and associated transcriptomics have been developed to investigate the genetic complexity in *B. napus* [110–111]. The GWAS and RNA-seq analyses of 505 inbred lines identifies hundreds of genes associated with seed oil content of *B. napus* and experiments of the homologous gene pair of *BnPMT6s* demonstrates that they negatively regulate seed oil content [42]. Meanwhile, homoeologous exchanges in allopolyploid genomes (AACC and AABB) were investigated by mRNAseq-based visualization [64]. Moreover, to reveal the rule of evolution in plant, a study has been conducted to explore the origin and diversification of *B. napus* using the comprehensive RNA-seq and organellar data [112].

To increase the resolution of gene expression profiling of specific tissue, laser capture microdissection (LCM) technology has been applied to isolate cell populations for RT-PCR or bulk sequencing [113, 114]. The study of papilla cell-expressed genes from *A. thaliana*, *Arabidopsis halleri*, and *B. rapa* by LCM coupled with RNA-sequencing (LCM-seq) identified specific genes involved in plant reproduction and development [115]. In *B. napus*, the distinct transcriptional feature expression of genes among the epidermis, cortex, and vasculature cells of the funiculus organ were analysed using LCM-seq, which revealed the coordination of these tissue systems to support seed development [116]. The comparative analysis of radish root-tissue- and stage-specific transcriptomes that were generated by LCM-seq and the previously reported transcriptomic data of *Arabidopsis* roots identified the evolutionary conserved stress-response gene-regulatory network; the network analysis identified that ERF-1 may be the novel key regulator of cambial activities [117]. LCM-seq enabled gene expression analysis with a higher spatial resolution than bulk RNA-seq. The drawback of LCM-seq is that a more limited number of cells can be analysed [113], and the difference between individual cells may remain unresolved after obtaining the average of gene expression profiles from cells [118].

The successful application of high-throughput single-cell transcriptome technologies in plant science in recent years has facilitated the discovery of the cell atlas at the single-cell level, providing new insights into cell heterogeneity and cellular function and helping us to understand the fundamental aspects of plant life [118–122]. These technologies are mainly classified into two types, namely single-cell RNA sequencing (scRNA-seq) and single-nucleus RNA sequencing (snRNA-seq). The droplet-based scRNA-seq approach includes three systems, namely inDrop, Drop-seq, and 10X Genomic Chromium [123]. The construction of scRNA-seq and snRNA-seq libraries relies on the isolation of protoplasts and nuclei, respectively. The major steps of data analysis include quality control, normalization, dimensionality reduction, cell type identification, and visualization [120–126]. The analytical results from both scRNA-seq and snRNA-seq offer opportunities to investigate plant cell identity and the function of tissues and organs [120].

For different tissues/organs of *A. thaliana*, including the roots [125–137], seedlings [138], the vegetative shoot apex [139], leaves [140–142], flowers [138], and seeds [143], the expression of genes at the single-cell resolution has been reported. These complex tissues/organs consist of diverse cell types. In *A. thaliana*, expressed genes from nine, eight, seven, ten, and five major cell types were identified in the roots, vegetative shoot apex, leaves, flowers, and seeds, respectively (Table 1). Some cell types can be further divided into several sub-cell types, such as four sub-cell types (pericycle, procambium, phloem, and xylem) for stele cells in the roots (Table 1). For each cell type, single-cell transcriptomic analysis provides cell type-specific expression genes, known as marker genes, for cell type discovery. Typically, marker genes are expressed differently among cell types but do not demonstrate an absolutely cell type-specific expression [119]. For each cell type, trajectory methods provide the possibility of refining cell-type identification and cell developmental transitions, such as the detection of protoxylem and metaxylem for xylem cell lineages [133]. Meanwhile, single-cell analysis provides an opportunity for inferring transcriptional factor regulatory networks, thereby elucidating the genetic coordination among cells [127–140].

**Table 1:** Discovery of cell types in *Arabidopsis thaliana* by high-throughput single-cell transcriptome analysis.

**Tissues/Organs**	**Major cell types**	**Sub-cell types**
Roots	(1) root cap cell, (2) trichoblasts (i.e. root hair cell), (3) atrichoblast (i.e. non-hair cell), (4) columella, (5) cortex, (6) endodermis, (7) stele cell, (8) quiescent center, (9) meristematic cell	(7) stele cell: (7.1) pericycle, (7.2) procambium, (7.3) phloem: (7.3.1) phloem procambium, (7.3.2) sieve element, (7.3.3) companion cell. (7.4) xylem: (7.4.1) protoxylem, (7.4.2) metaxylem
Vegetative shoot apex	(1) mesophyll cell, (2) shoot meristematic cell, (3) epidermal cell, (4) proliferating cell, (5) vascular cell, (6) guard cell, (7) companion cell, (8) shoot endodermis	—
Leaves	(1) mesophyll cell, (2) epidermis, (3) guard cell, (4) hydathode, (5) vascular cell, (6) meristemoid cell, (7) pavement cell	(5) vascular cell: (5.1) bundle sheath, (5.2) xylem, (5.3) phloem, (5.4) procambium, (5.5) companion cell
Flowers	(1) meristem, (2) anther, (3) perianth, (4) internode, (5) vasculature, (6) epidermis, (7) carpel, (8) mesophyll, (9) inflorescence axis, (10) stigma	—
Seeds	(1) peripheral endosperm (2) micropylar endosperm, (3) chalazal endosperm, (4) embryo proper, (5l) seed coat	(5) seed coat: (5.1) chalazal seed coat, (5.2) general seed coat

Studies on *A. thaliana* at the single-cell level provide opportunities and challenges for future studies on other species in Brassicaceae. The reported pipelines can be adopted for performing single-cell transcriptomic analysis. The reported marker genes in *A. thaliana* could be used to annotate cell types in other species by inferring the expression of their orthologs. However, several challenges remain, which are as follows: (i) orthologs of *A. thaliana* marker genes may not be conserved in other species [144], which may require experiments such as RNA *in situ* hybridization to confirm the discovery of cell types; (ii) the novel or rare cell types that exist in other species may be difficult to uncover, as no suggestion can be inferred from the previously reported cells; and (iii) the prior knowledge of non-model plants may be limited.

For complex tissues/organs in plants, their architecture is linked to biological function. Although a single-cell transcriptome provides the expression of genes at the single-cell level, the precise locations (i.e. the spatial contents) of the cells or tissues are lost. The development of spatial transcriptome technology enables the exploration of tissue architecture [145]. The single-cell spatial transcriptome of *A. thaliana* leaves can identify upper and lower epidermal cells, as well as the spatial developmental trajectories of vascular cells and guard cells [146], which provides opportunities to identify cells with limited knowledge of marker genes and to explore cell interactions and communications.

## Integration of Brassicaceae genomic information

With the enormous amount of sequencing data becoming publicly available in the post-genomics era, the development of tools for the efficient use of these sequencing data has become a pertinent research direction, with databases remaining one of the best tools. In recent years, in addition to the well-known Arabidopsis Information Resource, TAIR [147], many excellent databases have been developed for non-model Brassicaceae species, such as the Brassica Database (BRAD) [148], *B. napus* Pan-genome Information Resources (BnPIR) [149], gene expression database for *Brassica* crops (BrassicaEDB) [150], and Genomic Variation Database of *B. napus* (BnaGVD) [151].

The Brassicaceae database BRAD contains 36 reference genomes from 26 species [148]. It generates a table of syntenic genes of all genomes based on *A. thaliana* and *B. rapa* that is made available to the user. New features have been added such as the phylogenetic tree and sequence alignment of syntenic genes, homology comparison between two genomic fragments, primer design, retrieval of variant loci, and genomic sequence retrieval.

The developing knowledge system for Brassicaceae BrassiBase [152] includes cross-referenced information on the accurate enumeration of all species, genera, and tribes, chromosome numbers, genome sizes, morphological characteristics, and biological traits. The *B. napus* pan-genome information resource BnPIR [149] is a comprehensive database constructed based on the *B. napus* pan-genome (eight reference genomes) and 1688 rapeseed resequencing data. It was also the first pan-genome database for Brassicaceae crops, with GBrowse synteny and the pan-genome browser as key tools for using pan-genome data. The gene expression database for *Brassica* crops BrassicaEDB [150], a database focusing on gene expression in *Brassica*, specifically provides transcriptomic data and expression information for genes of *B. napus*. The genomic variation database of *B. napus* BnaGVD [151] specifically includes 34 591 899 high-quality SNPs and 12 281 923 high-quality InDels. It also provides tools for extracting annotations across 1007 accessions of worldwide rapeseed germplasm. These genomic databases that integrate omics data have facilitated studies on Brassicaceae species in the post-genomic era. The increasing amount of publicly available omics data will provide the opportunity for developing databases such as PLAZA [153] and Plant-ImputeDB [154] for comparative genomics and genotyping studies on Brassicaceae species.

Although these genomic databases have greatly increased the efficiency of the usage of genomic and other omics data, there are few tools for integrating genomic data with phenotypic data, which would greatly facilitate our breeding efforts. The GWAS Atlas database contains genotype–phenotype associations for understanding the genetic architecture of traits in *B. napus* [155], though very limited phenotypic data are available. Databases providing comprehensive phenotype information with powerful analysis tools for other Brassicaceae species are necessary.

## Future directions and prospects

Advances in genomics technologies have greatly promoted investigations into the genomes of Brassicaceae species. Population genomics will benefit from the greatly reduced cost for sequencing data production. It is now possible to resequence at a population scale of thousands or even over ten thousand accessions. Such studies will allow more accurate and reliable identification of genes associated with traits for Brassicaceae crops with much higher sensitivity. Pan-genomes of Brassicaceae crop species will be constructed to near completion with large numbers of representative accessions. Evidently, greater effort is still needed to integrate the rich genomic information with phenomic and metabolomic data to dissect interesting traits. Therefore, deep learning will play an important role in genomic interpretation of Brassicaceae crops in the future.

The combination of sequencing technologies with small-scale sample indexing, investigation of gene expression, genome modification, and 3D genome interaction is facilitating single-cell analysis. The newly developed spatial transcriptome analysis has a resolution at the scale of a few micrometers. Such a sub-cell scale analysis of gene expression provides us with a powerful tool for resolving the gene functions and networks of gene regulation. Emerging applications of these new technologies in *A. thaliana* are fascinating, and we are expecting digital 3D gene expression maps of different developmental stages at the cell level in Brassicaceae species.

Protein structure information greatly facilitates the resolution of gene functions. Recently, with the dramatic development of deep learning, the accurate prediction of protein structures became possible. DeepMind released the AlphaFold2, which has a protein structure prediction accuracy that competes with experimental structures in most cases and greatly outperforms other methods [156]. DeepMind and EMBL-EBI developed the AlphaFold Protein Structure Database (https://alphafold.ebi.ac.uk), which provides 992 316 protein structures, including *Arabidopsis* and *B. napus* proteins. The Baker lab at the University of Washington developed RoseTTaFold, which is slightly less accurate than AlphaFold2, but 100 times faster than AlphaFold2 and has lower hardware requirements [157]. RoseTTaFold has made it practical to build comprehensive protein structure databases for important *Brassica* species.

Based on the original motivation to study crop genomes, the achieved knowledge will push the genetic improvement of crops. Some important mechanisms unraveled by the investigation of trait domestication in *Brassica* species that are featured with duplicated genomes are of important value for directing breeding programs. During the trait domestication of *B. rapa* and *B. oleracea*, some of the homoeologous genes were selected in parallel [28]. This mechanism allowed us to propose a molecular design strategy on ‘combined selection of homoeologous genes’ for the genetic improvement of complex traits in polyploid or paleopolyploid species. Combining specific homoeologous genes significantly improved bolting tolerance and anti-cancer sulforaphane content in *B. rapa* [158–159]*.* We expect extensive application of this strategy to explore homoeologous genes.

Moreover, utilizing the wild and distant species is an important practice to introduce valuable alien genetic variation or genes into cultivated crops. Cheng *et al.* proposed a multi-vertex model to describe the possibility of crossing different Brassiceae species, which experienced the same genome triplication as *Brassica* [160]. This model provided a framework for utilizing the wild and distant species within the Brassiceae tribe. However, to facilitate successful utilization of the alien genomes, it is important to obtain high-quality genome sequences for as many Brassiceae species as possible.

Overall, with the dramatically fast development of sequencing technologies and bioinformatics, the investigation of Brassicaceae species is becoming increasingly intensive. The achieved knowledge will ultimately promote the breeding of Brassicaceae crops.

## Acknowledgements

This work was funded by the National Key Research and Development Program of China (2021YFF1000101), the Central Public-Interest Scientific Institution Basal Research Fund (Y2020PT21), and the Agricultural Science and Technology Innovation Program (ASTIP). This research was conducted in the Key Laboratory of Biology and Genetic Improvement of Horticultural Crops, Ministry of Agriculture, P.R. China and the Sino-Dutch Joint Lab of Horticultural Genomics Technology, Beijing.

## Author contributions

J.W. organized the manuscript and drafted and revised the manuscript with J.L. and R.L.; X.C., L.Z., X.G., T.W., and H.C. contributed to the collection of the published multi-genomics data of Brassicaceae genomes; X.W. organized and revised the manuscript. All authors read and approved the final manuscript.

## Conflict of interests

The authors declare that they have no conflict of interest.

## Supplementary data

Supplementary data is available at *Horticulture Research * online.

## Supplementary Material

Web_Material_uhac182
